# Endocytic CEACAM receptor loss and lack of positive selection in CEACAM1 of birds and snakes indicate absence of pathogen-CEACAM1 interactions in some reptiles

**DOI:** 10.21203/rs.3.rs-9693666/v1

**Published:** 2026-06-11

**Authors:** Wolfgang Zimmermann, Robert Kammerer

**Affiliations:** 1 Tumor Immunology Laboratory, LIFE Center, Department of Urology, University Hospital, Ludwig-Maximilians University, Munich, Germany; 2 Institute of Immunology, Friedrich-Loeffler-Institut, Federal Research Institute for Animal Health, Südufer 10, 17493 Greifswald - Insel Riems, Germany.

**Keywords:** carcinoembryonic antigen, turtle, crocodile, lizard, ITAM, ITIM, pathogen receptor, evolution

## Abstract

The CEACAM gene family is among the fastest evolving in vertebrates. It likely originated from an ancestral *CEACAM1*-like gene encoding an inhibitory receptor. In mammals, CEACAM1 is expressed on epithelial cells and can regulate leukocyte activation. Thus, several bacterial pathogens exploit it for host entry and immune suppression. Probably this threat selected for the evolution of decoy receptors with CEACAM1-like pathogen-binding domains. In humans, the granulocyte-specific decoy receptor CEACAM3 redirects CEACAM1-binding pathogens to neutrophils, promoting their uptake and destruction via its endocytic motif. Whether this evolutionary pattern occurs across all vertebrate clades remains unclear. Here, we identified CEACAM families in more than 100 reptile and 244 bird species. Non-avian reptiles such as turtles and crocodiles possessed expanded CEACAM gene families. Alongside the conserved *CEACAM19*, found in nearly all mammals, all non-avian reptiles carried *CEACAM1* and variable numbers of *CEACAM1*-related genes, mostly encoding transmembrane CEACAMs with endocytic ITAM motifs. Notably, snakes lacked these members. In contrast, some bird clades contained only a single CEACAM gene (*CEACAM1*), while others lacked *CEACAM* genes entirely. The absence of both decoy receptors and positive selection of *CEACAM1* in birds and snakes suggests these clades may lack CEACAM1-binding pathogens.

## Introduction

In humans, carcinoembryonic antigen-related cell adhesion molecule (CEACAM) decoy receptors with highly similar pathogen adhesin binding regions are part of the innate immune system. They counteract the use of CEACAM1 as entry receptor in epithelial cells and prevent downregulation of the immune response through engagement of the CEACAM1 receptor on T cells^1,2^. Inhibitory signaling via CEACAM1 engagement through adhesins presented on bacterial pathogens is mediated by the immunoreceptor tyrosine-based inhibition motif (ITIM). This has been demonstrated in human CD4-positive T cells for outer membrane vesicles from *Neisseria meningitidis* containing opacity associated (Opa) adhesins^3^. Numerous pathogens use CEACAM1 as entry and immune inhibitory receptor in humans including bacterial species within the genera *Neisseria, Moraxella, Haemophilus, Helicobacter, Streptococcus, Fusobacteria,* uropathogenic and enterotoxigenic *Escherichia, Acinetobacter* as well as viral and fungal pathogens such as *Candida*^4–16^. In humans, the granulocyte-specific CEACAM3 decoy receptor directs CEACAM1-targeting pathogens to neutrophils and initiates engulfment mediated by an immunoreceptor-based activation motif (ITAM) endocytic motif followed by pathogen destruction^1^. Such pairs of closely related receptors containing ITIM or ITAM motifs in their cytoplasmic domains are also called paired receptors^17^. Due to the short life cycle of pathogens, individual bacteria arise rapidly which can discriminate the entry receptor CEACAM1 from the endocytic receptor CEACAM3 and thus avoid destruction in the host^18^. On the other hand, human hosts with decoy receptor single nucleotide coding variants which make the pathogen-interacting region of the decoy receptor more similar to that of the entry receptor appear to have a reproductive and/or survival advantage^2,19^. Adjustment of receptor and decoy receptor sequences counteracting escape from immune surveillance happens fortuitously by gene conversion followed by Darwinian selection. Such events have been observed in amphibian, primate and other mammalian *CEACAM* gene families^2,17,20,21^. This suggests that CEACAM1 was appropriated by pathogens as entry receptor multiple times during evolution^2,20^.

Widespread occurrence of *CEACAM1* genes in vertebrates including cartilaginous and teleost fishes, amphibians and mammals indicates that it has essential functions in vertebrates^17,20,22,23^. Multiple functions have been identified for CEACAM1, like homotypic adhesion, tissue homeostasis, regulation of insulin signaling and immune regulation^24,25^.

However, no *CEACAM1* genes have been reported to exist in reptiles and birds although they share a common ancestor with fishes and amphibians. Therefore, to solve the conundrum of a possible gap of *CEACAM1* and related genes in a large vertebrate clade we searched nucleotide and amino acid sequence databases for the presence of *CEACAM*-related genes in more than 100 reptile and over 340 bird species. A large variety of *CEACAM* genes could be identified in non-avian reptiles whereas a large fraction of bird species appears to have lost all *CEACAM* genes with only a smaller fraction retaining a functional *CEACAM1* gene.

## Methods

### Identification and nomenclature of genes

Genome-wide nucleotide and amino acid sequence searches were performed using the NCBI blastn and blastp tools (http://www.ncbi.nlm.nih.gov/BLAST), the Ensembl (http://www.ensembl.org/index.html) and Whole-genome contigs shotgun (wgs) databases with default parameter setting. The accession numbers and genome coverage of the analyzed genomes as well as the species names and three letter identifiers are listed in Supplementary Tables 1 and 2. For identification of reptile and bird CEACAM genes initially human *CEACAM* N, transmembrane (TM) and cytoplasmic domain exon nucleotide sequences were used for the search. Reptile genomes were reprobed with exon sequences from newly identified reptile *CEACAM* and bird *CEACAM1* genes to identify additional related genes within the *CEACAM* gene family. N, TM and cytoplasmic domain exons of the most closely related reptile or bird species were used for the BLAST search applying the “discontiguous megablast” or “blastn” setting. When available, predicted complete CEACAM cDNA sequences identified in the NCBI core nucleotide database through hits with N, TM and cytoplasmic domain exon nucleotide sequences were used to identify and localize additional exons in related genes and species. Orthologous *CEACAM* genes were identified by synteny and/or by the presence of unique exons (e.g. exons encoding ITIM/ITSM motifs) using sufficiently large contigs and exon sequences from the most closely related species. *CEACAM* genes were numbered arbitrarily except for *CEACM1* and *CEACAM19*. Genes flanking the CEA loci in selected bird species were visualized using NCBI genome data viewer (https://www.ncbi.nlm.nih.gov/gdv/browser/genome/) and captured as screenshots. Simple sequences and transposable elements were identified with the Dfam data base (https://www.dfam.org). The completeness of the genomes can be estimated from the BUSCO metrics (https://busco.ezlab.org) which have been retrieved for a set of reference bird genomes (Supplementary Figure 1) as well as from the fold genome coverage where larger numbers indicate higher completeness of genomes (Supplementary Tables 1 and 2). Genome coverage details can be obtained from “Blast search databases information”. Support for domain organizations and differential splicing of CEACAM1 was obtained through blast searches in the Transcriptome Shotgun Assembly (TSA) database. Genes that contained stop codons within their N domain exons were considered to represent pseudogenes. Putative bird *CEACAM1* genes without transmembrane and ITIM/ITSM-encoding exons were assumed to represent incomplete genes. However, presently it cannot be excluded that such genes encode soluble CEACAMs. This point could be clarified with the aid of mRNA expression data. Such sequences, however, are lacking for most bird species. Nucleotide sequences from the N domain exons can be used as gene identifiers. These sequences and additional CEACAM exon sequences have been uploaded to the Zenodo open repository (https://zenodo.org) and can be retrieved using the Digital Object Identifier (DOI) https://zenodo.org/records/18457828?preview=1&token=eyJhbGciOiJIUzUxMiJ9.eyJpZCI6IjU0OThmZTMxLTYxYTktNGIxZi04ZWEyLWM0YmUxZjIwMDJlNCIsImRhdGEiOnt9LCJyYW5kb20iOiJlMzQzYjQ4NjdiODJlOGQwMjJhMzZjNTU2YzA4ODM5NSJ9.yix2tzktwO6dzwJ8ABnORz1V7cvWypEzZeFMKAaF8RlWTQgjr2EMvOMUrh5gMOr5onOApkQmM0mWXPPR0P3lDg.

### 3D modeling and visualization

3D model prediction was done on the AlphaFold 3 server (https://alphafoldserver.com)^26^. Molecular graphics and analyses were performed with UCSF ChimeraX (https://www.cgl.ucsf.edu/chimerax/), developed by the Resource for Biocomputing, Visualization, and Informatics at the University of California, San Francisco, with support from National Institutes of Health R01-GM129325 and the Office of Cyber Infrastructure and Computational Biology, National Institute of Allergy and Infectious Diseases.

### Phylogenetic analyses and determination of positive and purifying selection

Pairwise and multiple sequence alignments were performed with CLUSTALW programs (http://npsapbil.ibcp.fr/cgi-bin/npsa_automat.pl?page=/NPSA/npsa_clustalw.html; http://www.genome.jp/tools/clustalw/). Phylogenetic analyses based on nucleotide sequences were performed using MEGAX^27^. Sequences were aligned using MUSCLE ^28^ implemented in MEGAX. Phylogenetic trees were constructed using the maximum likelihood (ML) method with bootstrap testing (100 replicates). The best fit substitution model was selected within MEGAX. The evolutionary pressure driving diversification of nucleotide sequences was determined by calculating the number of nonsynonymous nucleotide substitution per nonsynonymous site (dN) and the number of synonymous substitutions per synonymous site (dS) for CEACAM N domain exons known to encode ligand-interacting and bacterial adhesin-binding domains. The dN/dS ratios between all pairs of CEACAM orthologs (i.e. the N exon of a given gene from each species was compared with the N exons of all other species) were calculated after manual editing of sequence gaps (filled in with Ns) or insertions (inserted nucleotides were removed) in aligned nucleotide sequences guided by the corresponding amino acid sequences using the Synonymous Nonsynonymous Analysis Program (SNAP) program (http://www.hiv.lanl.gov/content/sequence/SNAP/SNAP.html)^29^. The dS values were below saturation (i.e. less than 2) for all reptile groups. The accumulation of average synonymous and nonsynonymous substitutions along the N exons were visualized using the XPLOT function of that program. The number of reptile species used for both analyses is indicated in the figures and in the legends. The program PipMaker (http://bio.cse.psu.edu/) was used to identify conserved contiguous stretches of nucleotides between gene pairs and to calculate the degree of identity which is summarized as a ‘percent identity plot’^30^. This program compares each nucleotide sequence region of gene one with all regions of the second gene using a sliding window. Related sequences within a gene, like the multiple immunoglobulin constant-like (IgC) domain-encoding exons in the *CEACAM1* gene, result in several stacked parallel lines indicating the varying degree of similarity between repeated sequences.

### Statistical analysis

Statistical analyses were performed using GraphPad prism version 9.5.1. A P value < 0.05 was considered to represent a statistically significant difference.

### Use of Large Language Models

We used ChatGPT to help to correct syntax and grammar of the manuscript.

## Results

### Reptiles and birds harbor CEACAM family genes varying greatly in number and structure

Previously, we and others have identified *CEACAM* gene families in almost all vertebrate groups including mammals, amphibians and fishes except reptiles and birds. Here we close this gap by analyzing more than 100 reptilian and 340 avian species for the presence of CEACAM members (Supplementary Table1, 2).

**Figure 1 –:**
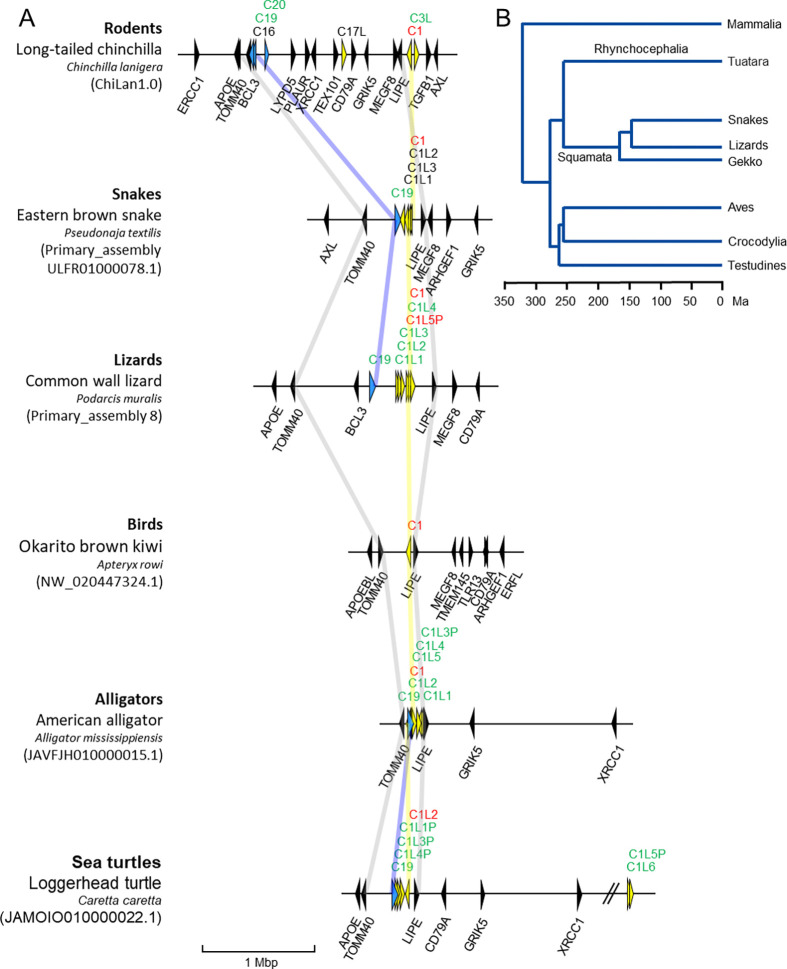
CEACAM-related genes in reptiles and birds exhibit synteny with rodent CEACAM loci. (A) The chromosomal arrangement of *CEACAM* genes in snake (Eastern brown snake), lizard (common wall lizard), bird (Okarito brown kiwi), alligator (American alligator) and sea turtle (loggerhead turtle) is shown and compared with the rodent chinchilla *CEACAM* locus. Arrowheads represent genes with their transcriptional orientation: *CEACAM1*-related *CEACAM* genes in yellow, orthologous *CEACAM* genes in blue, selected flanking genes in black. The CEACAM gene loci were aligned along the position of *CEACAM1* (yellow line), the *CEACAM19* loci are connected with a blue line, two conserved flanking genes (*TOMM40* and *LIPE*) by gray lines. Names of *CEACAM1*-like genes with ITIM/ITSM-encoding exons are shown in red and with ITAM motif-encoding exons in green. The scaffolds and databases used are indicated below the species name. C, CEACAM; C1L, CEACAM-like; P, pseudogene; Mbp, million base pairs. (B) Phylogeny of mammals and reptile groups including turtles (testudines), crocodiles (crocodylia) and birds (aves). Ma, million years. Adapted from Gemmell et al., 2020^49^.

We identified CEACAM gene loci by genome-wide searches of nucleotide databases using initially human exon sequences encoding immunoglobulin variable (IgV)-like, TM and ITAM-containing cytoplasmic domains from *CEACAM19,* a conserved member of the *CEACAM* gene family, and exon sequences encoding IgV-, IgC-like, TM and ITIM/ITSM-containing cytoplasmic domains of *CEACAM1*. We found *CEACAM*-related genes in reptiles and birds at locations syntenic to mammalian *CEACAM* loci (Figure 1).

**Figure 2 -:**
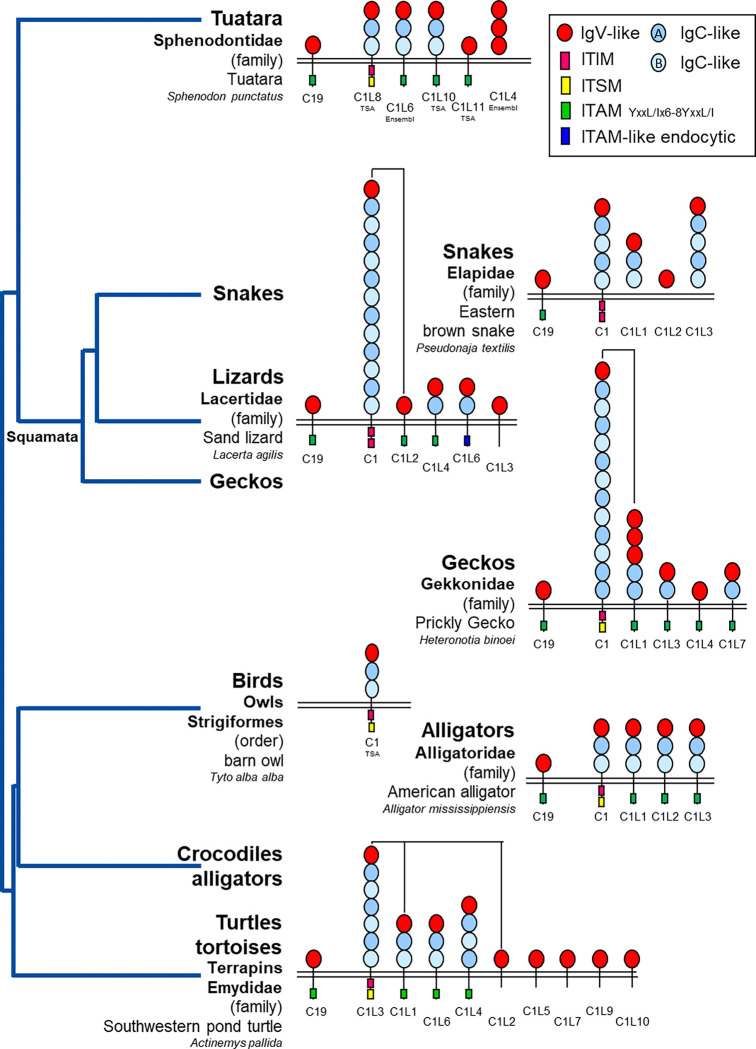
Domain organization of reptile CEACAM family members. The graphs were ordered based on their phylogenetic relationship indicated by the phylogenetic tree at the left margin (adapted from Gemmell et al., 2020 ^50^). The domain organization of CEACAMs from selected reptile species was predicted by gene analysis. Family and species names are indicated at the left side. Domain organizations were confirmed by expression data from TSA data bases when available. IgV-like domains are shown as red, and IgC-like domains as blue ovals. ITIM and ITSM motifs are indicated by red and yellow, ITAM and ITAM-like endocytic motifs by green and blue boxes, respectively. CEACAMs with closely related IgV domains possibly representing paired receptors are connected by brackets. C, CEACAM; TSA, Transcriptome Shotgun Assembly.

However, *CEACAM16*, *CEACAM18* and *CEACAM20*, three *CEACAM* genes well conserved in placental mammals and marsupials could neither be identified in reptiles nor in birds using the respective human or murine N exon nucleotide sequences for the database search. The alligator (*Alligator mississippiensis*) and the loggerhead sea turtle (*Caretta caretta*) *CEACAM* loci exhibited a more compact arrangement compared to snake and lizard gene loci as can be seen by the distance of flanking non-*CEACAM* genes (*TOMM40*, *LIPE*) in the alligator and eastern brown snake as well as common wall lizard genomes (Figure 1). This is probably due to much smaller introns in these as well as in bird species compared to those found in lizards and snakes (exemplified for *CEACAM1* in Supplementary Figure 2). In addition, similar to the South American rodent chinchilla, most of the reptile *CEACAM* gene clusters lack genes encoding secreted CEACAM-related pregnancy-specific glycoproteins (PSG)-like proteins present in mammals with hemochorial placentae ^31,32^ with the possible exception of snakes (*CEACAM1L1, CEACAM1L2, CEACAM1L3*), tuatara (CEACAM1L4) and the painted turtle (*CEACAM1L1 and CEACAM1L2*) (Figure 2; Supplementary Figure 3C, F).

Interestingly, in the snake soluble CEACAM1L1 protein disintegrin-like motifs as found in human PSGs were identified slightly more COOH-terminally in the majority of snake species (Supplementary Figure 4A-C). Such RGD-related tri-peptide motifs may function like snake venom disintegrins, which bind integrins and inhibit interactions with corresponding ligands ^32,33^. Furthermore, the *CEACAM* gene locus is not interrupted by long genomic regions with non-*CEACAM* genes as seen for example for the rodent *CEACAM* gene locus (Figure 1).

**Figure 3 -:**
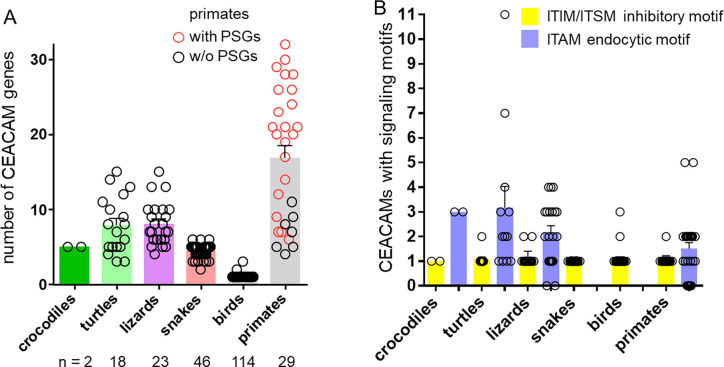
Size of *CEACAM* gene families of reptiles, birds and primates. (A) Number of *CEACAM-*related genes (mean – SEM) in alligator, turtle, lizard, snake, bird and primate species (number of analyzed species are indicated below the graph). In primates with *PSG* genes, the numbers of *CEACAM*-like genes are shown with red symbols, in primates without *PSG* genes with black symbols. (B) Number of *CEACAM*-related genes (mean – SEM) with ITIM/ITSM motifs and ITAM endocytic motifs are shown in yellow and blue, respectively. The ITAM-encoding *CEACAM19* genes are not included in the count. ITAM, immunoreceptor tyrosine-based activation motif; ITIM, immunoreceptor tyrosine-based inhibitory motif; PSG, pregnancy-specific glycoprotein; immunoreceptor tyrosine-based inhibitory motif; ITSM, immunoreceptor tyrosine-based switch motif.

In the various groups of reptiles, we found on average between 5–9 *CEACAM* genes similar as the primate group that lack CEACAM-related *PSG* genes (Figure 3).

The encoded proteins are predicted to exhibit considerable variation in domain organization. Most of them are composed of one immunoglobulin variable (IgV)-like domain or N domain and a highly variable number of IgC-like domains ranging from 0 to 16 domains (Figure 2; Supplementary Figure 3). Some CEACAM proteins are predicted to contain several IgV-like domains in tandem either composed entirely of IgV-like domains like the tuatara CEACAM1L4 consisting of three N domains or multiple IgV- and IgC-like domains as found in the prickly gecko (*Heteronotia binoei*) transmembrane bound CEACAM1L1 which contains three N-terminal N domains followed by two IgC-like domains (Figure 2). The N domain-encoding exon also codes for the last third of the leader. Therefore, mutational inactivation of this exon abolishes expression of the corresponding CEACAM gene.

In contrast, birds have at most a single functional *CEACAM* gene (*CEACAM1*). Apparently intact *CEACAM1* genes could be identified in members of the superorder *Palaeognathae* which encompass kiwis, cassowaries and ostriches, and in the *Aequornithes* clade comprising flamingos, plovers, penguins, puffins and cormorants as well as in the *Accipitriformes* order which includes hawks, eagles and vultures. No complete CEACAM genes were found in water and land fowl (superorder *Galloanserae*) with the most prominent members ducks and chicken as well as in song birds (*Passeriformes*) (Supplementary Figure 5). To estimate the sensitivity of detection we used the *CEACAM1* N exon nucleotide sequence of the Okarito brown kiwi (*Apteryx rowi*) to identify the *CEACAM1* N exon in the most distantly related bird species with *CEACAM1* based on the phylogenetic tree shown in Supplementary Figure 5 i.e. the black-legged seriema (*Chunga burmeisteri*). We could identify a nucleotide sequence with a query cover of 99% and 66% identity (E value 2e-17). Searches using *CEACAM1* TM and cytoplasmic tail exon 3 sequences (encoding the conserved ITIM/ITSM motifs) yielded hits with query cover of 60% and 91.8% identity (E value 2e-11) and 88% and 74.3% (E value 6e-08), respectively.

We have analyzed the CEA gene family locus in a subset of birds to better understand why we did not find CEACAMs in all bird species. As shown in Supplementary Figure 1, the CEA gene family locus in birds is extremely small and contains only the *CEACAM1* gene flanked by the genes *LIPE* and *BCL3*. In species where we did not find complete *CEACAM1* genes we either detected defective *CEACAM1* exons (e.g. *Galloanserae*) or the complete CEA locus was missing, or replaced by *CEACAM* gene-unrelated sequences (Supplementary Figure 1) even in birds with high quality genomes with a BUSCO score > 97 %. As an example for defective *CEACAM1* exons, the nucleotide sequences of N exons from 13 different chicken breeds were aligned (Supplementary Figure 6). Multiple deletions (including a truncation of the 5’-region) and/or insertions can be seen which in most cases result in frame shifts and generation of stop codons (not shown).

To rule out that existing *CEACAM* genes were not found due to incompleteness of the analyzed genomes we compared the sequencing depth of the analyzed bird genomes with or without *CEACAM1* genes, based on the presence or absence of N exons. The median sequencing depth was lower in bird species without *CEACAM1* genes (n=103) in comparison to that of birds with *CEACAM1* genes (n=243; 84 versus 90-fold). However, this difference was not significant (P=0.4833, two-tailed Whitney U test).

In most reptile species only one *CEACAM1* gene was found; a few turtle/terrapin, lizard and bird species contain two or more *CEACAM1* genes. They contain either two ITIM motifs or one ITIM (consensus sequence in one letter code: S/I/V/LxYxxI/V/L) and one immunoreceptor tyrosine-based switch motif (ITSM; consensus sequence: TxYxxV/I) (Figure 2). Interestingly, in terrapins, like the southwestern pond turtle (*Actinemys pallida*), one to several *CEACAM1*-derived genes exist which are predicted to be composed of one N domain and a transmembrane domain, but lacking ITIM/ITSM signaling domains. This feature is caused by a mutation in the six-nucleotide consensus splice donor site (GTA/GAGT) at the 3’-end of the transmembrane domain-encoding exon (e.g. GgAAGc in *CEACAM1L7*, compared with GTAAcg in the *CEACAM1* ortholog *CEACAM1L3* with replacement of the otherwise highly conserved T by a G). This mutation may cause read-through into the intron sequence until a nearby stop codon is encountered, resulting in the exclusion of exons encoding cytoplasmic ITIM/ITSM domains (Figure 2). This group of *CEACAM1*-derived genes exhibits a high degree of nucleotide sequence similarity between their N exons (96.2–99.2% identity) and the N exon of the *CEACAM1* ortholog *CEACAM1L3* while the ITAM motif-bearing members (*CEACAM1L1*, *CEACAM1L4* and *CEACAM1L6*) show only between 85.9 and 91.4% identity in this species.

Reptile *CEACAM1* genes vary largely in the number of IgC-like domain encoding exons. This is especially obvious in lizard species where the number of IgC-like exons can vary from 4 in the Komodo dragon to 16 in the common wall lizard. Close relatedness of pairs of A- and B-type IgC-like domain-encoding exons indicate recent amplification of the corresponding genomic stretches (Supplementary Figure 7). Alternative splicing can lead to numerous mRNA variants as exemplified by the CEACAM1 splice variants found in the venom gland of the Western rattle snake (*Crotalus viridis*). Twelve CEACAM1 splice variants could be identified. The predicted CEACAM1 isoforms differ in the number of IgC-like domains (0, 2 ,3, 4, 5, or 6) and in the presence or absence of ITIM motifs caused by inclusion and exclusion of the 53-bp cytoplasmic domain exon 1, respectively (Supplementary Figure 8).

### Snakes and birds lack CEACAM family members with ITAM signaling motifs

Varying numbers of *CEACAM1*-related genes encoding ITAM endocytic motifs were identified in the tuatara, crocodiles/alligators, turtles/tortoises, geckos and lizards ranging from one in the central bearded dragon (*Pogonia vitticeps*) to 11 in the green sea turtle (Supplementary Figure 3C, D). The conserved *CEACAM19* ITAM-containing gene is not included in this count. The cytoplasmic tail with the ITAM motifs of the CEACAM1-related proteins are encoded by three exons in reptiles, which is in contrast to mammals, where four cytoplasmic domain exons are observed^17^ (Supplementary Figure 9). Interestingly, in most of the reptile species a single Y-containing motif conforming to a so-called endocytic motif is found between 17 and 21 amino acids upstream of the proximal Y of the ITAM motif. A subset of the upstream motif is also compliant with an inhibitory ITIM motif (Supplementary Figure 9). Snakes and birds lack ITAM-containing *CEACAM* genes altogether (Figure 2; Supplementary Figure 3).

### CEACAM1 N domains in crocodiles and turtles exhibit selection for diversification

**Figure 4 -:**
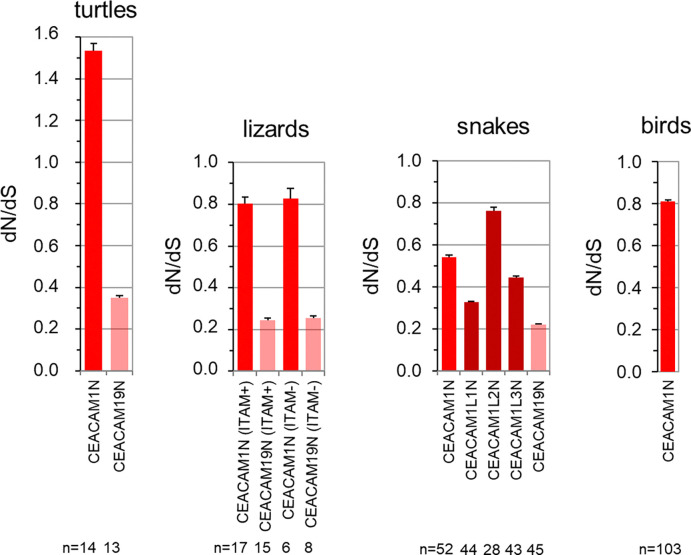
Darwinian selection for diversification is high in turtle *CEACAM1* IgV domain exons. dN/dS values between orthologous *CEACAM* genes were calculated for all pairwise combinations of *CEACAM* N exons using the SNAP program (see Methods section). Please note, that *CEACAM* genes with complete exon sequences could not be retrieved for all species from the databases. Genes with incomplete or missing N exons were excluded from the calculation. The number of genes used for the dN/dS calculation is indicated at the bottom of the graph. The results are displayed as mean values with standard error of the mean (SEM). Please note that the dN/dS ratios for CEACAM1 N in lizard species with or without ITAM-containing CEACAMs differ only marginally.

Selection for diversification is typical for pathogen receptors particularly in regions involved in pathogen adhesin interactions. This has been noted for the N domains of primate CEACAM pathogen receptors^2^. High ratios (>1) of rates of nonsynonymous and synonymous substitutions (dN/dS) between closely related species is indicative of such pressure exerted by pathogens. Calculation of dN/dS ratios for *CEACAM1* N exons using the SNAP program revealed a ratio of about 1.5 for turtles and ratios between 0.55 and 0.81 for crocodiles, lizards, snakes and birds (Figure 4).

**Figure 5 –:**
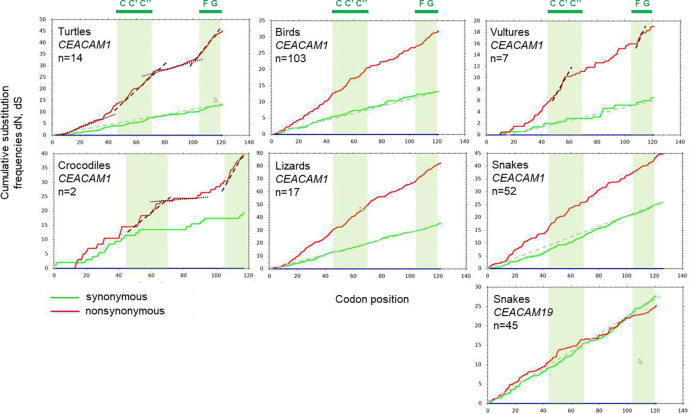
Selection for diversification in putative pathogen binding regions of *CEACAM1* in crocodiles, turtles and vultures. In crocodiles, turtles and vultures, nonsynonymous mutations accumulate preferentially in sections of *CEACAM1* N exons encoding ligand binding regions in the CC’C’’FG IgV β-sheet. The cumulative frequencies of nonsynonymous (dN; red curves) and synonymous substitutions (dS; green curves) along the N exons of orthologous *CEACAM1* genes from turtle (n=14), bird (n=103), vulture (n=7), crocodile (n=2), lizard (n=17) and snake species (n=52) were determined. For a subgroup of birds, i.e. vultures (n=7) cumulative substitution frequencies dN and dS were separately calculated. The low number of species for the crocodile/alligator clade is due to low genome sequence depth of additional four crocodile/alligator species. Only from two (American alligator, gharial) out of six available crocodile/alligator genomes (Chinese alligator, spectacled caiman, Cuban crocodile, Australian salt water crocodile) complete *CEACAM1* N exons could be retrieved. For comparison, dN and dS were calculated for the N exons of snake *CEACAM19* genes. Note the preferential accumulation of nonsynonymous mutations in the CC’C″FG β-strand regions (steeper black dashed lines) which indicates selection for diversification. This contrasts with conserved regions between CC’C″ and FG β-strands (dotted lines). The location of CC’C″ and FG β-strand regions is indicated by green shading in the graphs. Note the expected steady accumulation of synonymous substitutions indicated by gray dashed lines.

In contrast, *CEACAM19,* a conserved *CEACAM* gene found in all mammals and non-avian reptiles, which is not known to interact with pathogens, shows low N exon dN/dS ratios between 0.22 and 0.34 in various reptile groups (Figure 4). Interestingly, in snakes. additional genes (*CEACAM1L1, CEACAM1L2, CEACAM1L3*) encoding secreted proteins (Figure 2; Supplementary Figure 3) were found for which orthologues could be identified based on sequence similarity of their N exons within snakes. They exhibit dN/dS ratios between 0.33 and 0.76, whereby CEACAM1L2 N exceeds the ratio of snake *CEACAM1* N exon (0.54; Figure 4). The SNAP program also allows to visualize the accumulation of non-synonymous mutations along the N exons. This analysis revealed in turtles that such mutations accumulate preferentially in the CC’C’’FG face of the IgV-like N domain of CEACAM1 which is known in other species to interact with bacterial pathogen adhesins and endogenous ligands^34^. In contrast, in lizards, snakes and birds a nearly even accumulation of non-synonymous mutations is found. This was also observed for the N exon of snake *CEACAM19* genes. Although the overall dN/dS ratio for two alligator/crocodiles *CEACAM1* N exons is quite low (0.70) a preferential accumulation in the ligand binding region especially that is formed by the FG β-strands is noticed (Figure 5).

Interestingly, although bird *CEACAM1* N exons when analyzed collectively from diverse groups of species did no exhibit a selective accumulation of nonsynonymous mutations in the putative pathogen binding region, a subgroup of birds i.e. vultures which are probably exposed to a huge array of pathogens due to their scavenger lifestyle showed a selective accumulation of nonsynonymous mutations in the CC’C’’FG β-sheet of the N domain (Figure 5). This resulted in a relatively high N exon dN/dS ratio of 0.98.

### Interspecies diversification of putative CEACAM pathogen receptor genes in crocodiles and turtles is restricted to N exon regions

**Figure 6 -:**
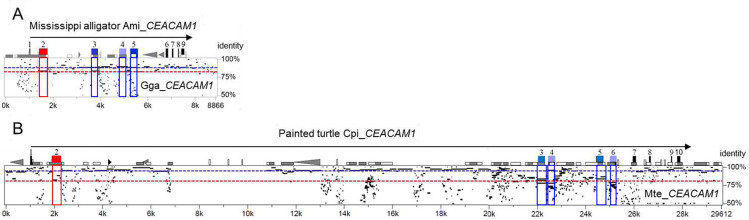
Interspecies diversification of N exon regions of putative pathogen *CEACAM1* receptor genes. Conservation of nucleotide sequences of *CEACAM1* genes from the American alligator and the crocodile species gharial (A) and the freshwater turtles painted turtle and diamondback terrapin (B) were analyzed (gene names and species acronyms are shown in the lower right corner of the plot) using the PipMaker program. For contiguous stretches of nucleotides conserved between gene pairs, the degree of identity was calculated and displayed as horizontal black lines. The location of exons of the gene listed on top of the graphs is indicated by numbered boxes (red, IgV domain-encoding N exons; blue, IgC domain-encoding exons, black, transmembrane and cytoplasmic domain exons). Repeat sequences/mobile elements are shown by differently gray colored and shaped forms. For comparison, the level of conservation of the N exon regions is marked by red dashed lines, that of the most conserved IgC exon by blue lines. Red- and blue-outlined boxes indicate N exon- and C exon-encompassing regions of common identity. Ami, *Alligator mississippiensis* (American alligator); Cpi, *Chrysemys picta bellii* (painted turtle); Gga, *Gavialis gangeticus* (gharial); Mte, *Malaclemys terrapin* (diamondback terrapin).

If CEACAM1 in reptiles is also highjacked by pathogens as a receptor to enter cells and to downregulate the immune response via inhibitory signaling in T cells as observed in humans^1^, one would expect most mutations to occur in the pathogen-binding N domain-encoding exons in *CEACAM1* genes and to result in amino acid changes. Such changes are likely positively selected particularly when they diminish pathogen adhesin/receptor interaction thereby conferring reproductive advantages for the individual and becoming fixed in the population. Indeed, comparison of full-length *CEACAM1* gene sequences of the alligator (*Alligator mississippiensis*) and the gharial crocodile (*Gavialis gangeticus*) as well as of the turtle species painted turtle (*Chrysemys picta bellii*) and diamondback terrapin (*Malaclemys terrapin*) revealed low conservation of N exons compared with other coding exons (see e.g. IgC-like B1 domain-encoding exon 4 conservation in turtles) and even compared with non-coding intron regions which are more highly conserved (Figure 6A, B).

This selective diversification of the N domain-encoding exons is also observed for *CEACAM1* of lizards such as the sand lizard and the common wall lizard (Supplementary Figure 10A) suggesting past or ongoing use as pathogen receptor in these species, despite a low overall dN/dS ratio for lizards *CEACAM1* N exons (Figure 4).

### N exon regions of CEACAM pathogen receptor and decoy receptor genes are most similar within reptile species

**Figure 7 -:**
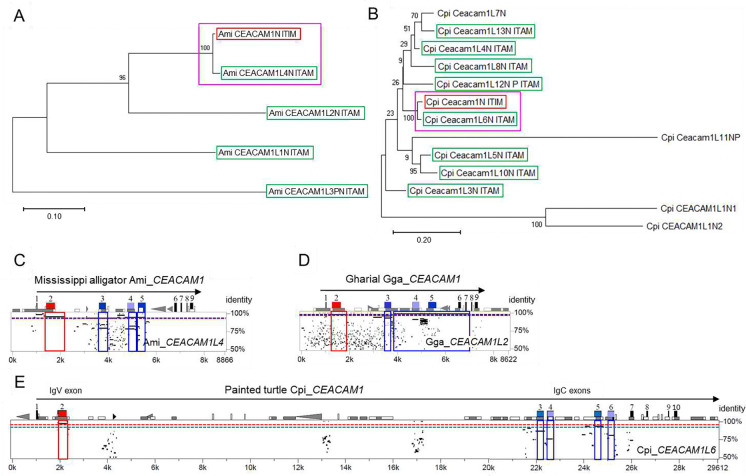
Intraspecies conservation of N exons between *CEACAM1* and putative pathogen *CEACAM* decoy receptor genes in reptiles. The phylogenetic relationship of N exon nucleotide sequences of *CEACAM* genes from American alligator (A) and the painted turtle (B) were analyzed using the Maximum Likelihood method implemented in the MEGAX program. The bars below the phylogenetic trees indicate the scale of the branches (number of substitutions per site). The numbers at the branching points of the phylogenetic tree represent Bootstrap values. ITIM and ITAM signaling motif-encoding genes are marked with red and green boxes, respectively. The genes with the most closely related N exons are boxed with purple lines. Conservation of nucleotide sequences of *CEACAM1* and *CEACAM1L4* genes from the American alligator (C), the *CEACAM1* and *CEACAM1L2* genes from the crocodile species gharial (D) and *CEACAM1* and *CEACAM1L6* from the painted turtle (E) were analyzed (gene names and species acronyms are shown in the lower right corner of the plot) using the PipMaker program. For contiguous stretches of nucleotides conserved between gene pairs, the degree of identity was calculated and displayed as horizontal black lines. The location of exons of the gene listed on top of the graphs is indicated by numbered boxes (red, IgV domain-encoding N exons; blue, IgC domain-encoding exons, black, transmembrane and cytoplasmic domain exons). Repeat sequences/mobile elements are shown by differently gray colored and shaped forms. For comparison, the level of conservation of the N exon regions is marked by red dashed lines, that of the most conserved IgC exon by blue lines. Red- and blue-outlined boxes indicate N exon- and C exon-encompassing regions of common identity. Please note the extended regions of high similarity between *CEACAM1* and putative decoy receptor genes in alligator and gharial, indicating recent gene conversion events. Ami, *Alligator mississippiensis* (American alligator); Cpi, *Chrysemys picta bellii* (painted turtle); Gga, *Gavialis gangeticus* (gharial).

This contrasts with the intraspecies conservation of the N exon nucleotide and encoded amino acid sequences between *CEACAM1* and the corresponding putative decoy receptors genes that encode endocytic motifs (Figure 7A, B). For example, comparison of painted turtle *CEACAM1* and its closest N exon relative, *CEACAM1L6* (Figure 7B) reveals high conservation of the N exon nucleotide sequence (95.8% identity), whereas IgC-like domain A and B type exons show much lower conservation: 85.5% and 93.1% identity for A1 and A2 and 73.8% and 74.9% identity for B1 and B2, respectively (Figure 7E).

Similarly, selective high conservation of N exon nucleotide sequences between *CEACAM1* and the putative decoy receptor *CEACAM1L1* is observed in the common wall lizard (Supplementary Figure 10B). High conservation of N exon sequences can extent into neighboring intron sequences, as seen in comparisons of *CEACAM1* with their respective closely related putative decoy receptor genes *CEACAM1L4* in Mississippi alligator and *CEACAM1L2* in the gharial (Figure 7C, D).

### Similarity of N exon sequences of *CEACAM1* and decoy receptor genes can be maintained by gene conversion

In crocodiles and alligators, *CEACAM1* and putative decoy receptor genes (*CEACAM1L4, CEACAM1L2*) exhibit opposite transcriptional orientation (Figure 1 and data not shown) which allows intrachromosomal recombination leading to gene conversion^2^. A similar arrangement is observed for turtle *CEACAM1* and putative decoy receptor genes in the green sea turtle (Figure 1) as well as in Western mud turtle and painted turtle (not shown). Gene conversion involves alignment of related exon sequences following intrachromosomal loop formation, allowing one sequence to be copied and replace the second sequence (Supplementary Figure 11A) ^35,36^. Exon-proximal sequences can also be involved provided they are sufficiently similar to form heteroduplexes which leads to extended sequence similarity flanking the affected exons, as seen in the alligator and gavial as well as in the lizard central bearded dragon (*Pogona vitticeps*) (Figure 7C, D; Supplementary Figure 11B). However, this mechanism does not appear to apply to the putative decoy receptors derived from ITIM-containing genes in the Southwestern pond turtle and painted turtle as these genes share the same transcriptional orientation as the corresponding *CEACAM1* gene.

## Discussion

In this study we identified *CEACAM*-related genes in reptiles including birds. This finding extends the presence of CEACAM to all major vertebrate clades, from mammals to cartilaginous fishes^17,22,23,37^. However, as shown here, major avian clades such as songbirds and fowl lack intact CEACAM genes entirely. This absence is due to gene loss, as the common ancestor of reptiles and birds must have harbored *CEACAM* genes in its genome inherited from earlier vertebrate lineages. Failure of detection of CEACAM genes in a significant fraction of bird species due to an insufficient screening protocol could be ruled out by several control experiments (see Result section).

In reptiles, all CEACAM genes are located adjacent to one another including the more distantly related yet highly conserved member *CEACAM19*. Expansion of gene families typically occurs through crossing-over events following illegitimate pairing between two related genes in close proximity on sister chromatids, resulting in amplification of neighboring genes that often share the same transcriptional orientation^36^. Reptiles, therefore, exhibit a more ancestral *CEACAM* gene organization whereas rearrangement in younger clades such as mammals led to separation of conserved *CEACAM* genes, including *CEACAM19* from *CEACAM1*-related genes by insertion of non-*CEACAM* genes^17^. Interestingly, reptiles lack *CEACM16* and *CEACAM20*, which are highly conserved in mammals. This suggests that these genes originated after the split of reptilian and mammalian ancestors namely, the sauropsids and the synapsids more than 300 million years ago^38^.

PSG probably evolved in mammals with hemochorial placentae to protect fetal cells from recognition as foreign by maternal immune cells, which come into direct contact with semi-allogeneic fetal tissues^39^. Maternal immune cells may also directly contact fetal cells in reptiles, particularly in viviparous species^40,41^. Notably, in all snakes, three out of a total of five *CEACAM1*-like genes encode secreted proteins, which may represent PSG-like proteins. Interestingly, in one of these secreted proteins (CEACAM1L1) RGD-related motifs - similar to those found in human and rodent PSGs^32^ - are present in the N domain of the majority of snake species although they are located more COOH-proximally. However, there appears to be no correlation with viviparity, as both viviparous snakes (e.g., vipers) and egg-laying snakes (e.g., pythons) carry RGD-motifs in CEACAM1L1. mRNAs for these secreted proteins have been identified for various snakes (many-banded krait (Bmu), South American coral snake (Mle), Amazon coral snake (Msp)) only in the venom gland, which is a preferentially investigated tissue in snakes for the identification of toxic proteins. Consequently, a reproduction-relevant expression pattern could not be inferred.

Acquisition of CEACAM1 as an entry receptor by pathogens, as documented in humans and likely also in other mammals and more distantly related vertebrates^2,19,20^ has been proposed as an important driver of *CEACAM* gene family evolution^42,43^. To date, CEACAM-binding pathogens have been identified only in humans and in mice^44,45^. Nonetheless, several observations are consistent with the possibility that CEACAM1 has been appropriated by pathogens in other species. These include (1) elevated dN/dS ratios in the exon encoding the pathogen-binding domain of the pathogen receptor among closely related species; (2) the presence of putative decoy receptors containing endocytic motifs and conserved pathogen binding regions; (3) gene family arrangement that allows maintenance of receptor and decoy receptor similarity through gene conversion and (4) indications of gene conversion between receptor/decoy receptor genes.

Turtles represent a reptilian lineage in which several of these features are observed. In particular, signatures consistent with diversifying selection are apparent in the N exons which encode the pathogen-binding domain in humans^19^, as indicated by dN/dS ratios exceeding 1 and by relatively low conservation of N exons of *CEACAM1* genes among closely related species that may be exposed to distinct pathogen challenges (Figures 4, 5 and 6). In addition, multiple CEACAMs containing endocytic motifs were identified that possess N domains closely related to the corresponding CEACAM1 N domain (Supplementary Figure 3C). In some turtle species (the Southwestern pond turtle and the painted turtle) single IgV domain *CEACAM1*-like genes were identified and are predicted to have lost expression of ITIM motif-encoding exons due to read-through of the transmembrane exon. The lack of inhibitory signaling combined with their high similarity with the CEACAM1 N domain raises the possibility that these proteins may act as decoy receptors, potentially diverting pathogens toward non-productive invasion pathways (Figure 2). If so, these CEACAMs would represent a previously unrecognized type of decoy receptor in the CEACAM family distinct from the ITAM-containing and GPI-linked decoy receptors proposed in primates^2^. The functional relevance of these predicted single-domain transmembrane CEACAMs in terrapins remains uncertain as expression data are currently lacking for the Southwestern pond turtle. Nevertheless, the opposite transcriptional orientation of *CEACAM1* and putative decoy receptor genes (Figure 1) is consistent with a genomic configuration that could allow gene conversion by intrachromosomal loop formation^2,36^. Such a mechanism could promote coordinated evolution of pathogen-binding domains in receptor and decoy receptor genes, potentially limiting pathogen discrimination between functional receptors and decoys. Consistent with this notion, signatures suggestive of gene conversion are observed in turtle CEACAM genes (Figure 7E). Several of the features discussed here are also present in alligators and crocodiles (Figures 4, 5, 6 and 7), suggesting that similar evolutionary dynamics may operate more broadly within reptilian lineages, although direct functional evidence is currently lacking.

Some or most indicators that have been associated with the acquisition of CEACAM1 as pathogen receptor such as the presence of candidate decoy receptors are absent in snakes and birds. The mouse family (*Muridae*) also lacks CEACAMs with endocytic ITAM motifs, despite the fact that mouse hepatitis virus (MHV) uses murine CEACAM1 as entry receptor in the house mouse (*Mus musculus*)^46^. In this case, evolutionary adaptation to pathogen pressure nonetheless appears to have occurred, as suggested by a high dN/dS ratio (> 1.3) for CEACAM1 N across mouse species^31^ and by the presence of *CEACAM1* alleles in wild mouse populations that do not bind MHV (Kammerer and Zimmermann, unpublished observations). Therefore, there could be a pathogen challenge in reptile species without endocytic motif-containing CEACAMs like snakes and birds. However, low dN/dS ratios observed for CEACAM1 in these clades make this scenario unlikely (Figure 4) possibly with the exception of birds with presumably heavy exposure to pathogens like vultures due to their dietary specialization (Figure 5).

Lizards also exhibit a relatively low dN/dS ratio (0.8) and no accumulation of non-synonymous substitutions in putative pathogen adhesin interacting regions within their N domains (Figures 4 and 5). However, there appears to be an evolutionary pressure leading to an unusual expansion of the extracellular region of CEACAM1 in some lizard families reaching up to 16 IgC-like domains (Supplementary Figure 6). This expansion of IgC-like exons may have been facilitated by larger intron sequences extended by high transposon activity in non-archosauromorph reptiles such as lizards and snakes^47^ possibly creating related sequence templates for amplification through recombination events. In contrast, in most mammals CEACAM1 contains 3–4 IgC-like domains, whereas in birds, crocodiles and turtles maximally 4–6 IgC-like domains are found (Supplementary Figure 3). This increase in domain number raises the question of whether it can be explained by selective pressure exerted by pathogens. In general, bacterial pathogens must bind to more than one receptor simultaneously to gain entry into host cells^48^. Extended extracellular regions formed by multiple IgC domains could reduce the ability of pathogens to simultaneously engage the distally located putative adhesion binding domain of CEACAM1 and non-CEACAM co-receptors on the cell surface, thereby limiting pathogen invasion. By contrast, putative paired decoy receptor pathogen binding sites on phagocytic cells do not need to be positioned far away from the phagocyte cell surface to effectively mediate pathogen engulfment, as exemplified by the single Ig domain-containing endocytic receptor CEACAM3 on human granulocytes^1^. These receptors contain only 0–2 IgC-like domains. The observed lack of selection for diversification in the CEACAM1 N domain in lizards (Figure 5) may therefore indicate that extended extracellular domains provide sufficient protection to prevent pathogen invasion superseding the need for diversification of pathogen-binding sites.

## Conclusion

Taken together, in crocodiles and alligators as well as in turtles multiple lines of evidence suggest acquisition of CEACAM1 as pathogen receptor. In contrast, loss of endocytic CEACAM receptors in snakes and bird species indicates absence of pathogen-CEACAM1 interactions in these lineages.

## Supplementary Material

This is a list of supplementary files associated with this preprint. Click to download.

• SupplementaryTable1Nonavianreptilegenomicdatasource.docx

• SupplementaryFigure6AlignmentofCEACAM1Nexonnucleotidesequencesfromchickenbreeds.docx

• SupplementaryFigure9ReptileandhumanCEACAMITAMmotifs.docx

• SupplementaryFigure11CEACAMNdomainaminoacidsequenceidentityandputativegeneconversionevent.docx

• SupplementaryTable2Birdgenomicdatasource.docx

• SupplementaryFigure2IntronsizevariationinreptileCEACAM1genes.pptx

• SupplementaryFigure5CEACAM1genepresenceinbirdgroups12.04.2026.pptx

• SupplementaryFigure7ExpansionofextracellulardomainsofCEACAM1inlizards.pptx

• SupplementaryFigure10SelectionforCEACAMNexondiversificationandconservationinlizards.pptx

• SupplementaryFigure8CEACAM1splicevariantsinWesternrattlesnake.pptx

• SupplementaryFigure3CEACAMdomainorganizationindifferentreptileclades.pptx

• SupplementaryFigure1CEAgenefamilylociofbirdswithandwithoutCEACAM1.pptx

• SupplementaryFigure4DisintegrinlikemotifsinsnakeCEACAMNdomains.docx

**Supplementary Figure 1 - CEA gene family loci in birds with or without *CEACAM1*.** Comparison of the CEA gene family locus in selected bird genomes where *CEACAM1* was found (A) and bird genomes where *CEACAM1* was not found (B). Screen shots of the NCBI genome graphic view show the sequence between the flanking genes *LIPE* and *BCL3*. Arrowheads indicate the direction of the transcription of the genes. The quality and completeness of the annotation is indicated by BUSCO analysis as provided by NCBI. (C) Analysis of transposable elements in the genomic region between *LIPE* and *BCL3* of the songbird *Luscinia svecica*. Besides various simple sequences (black lines) a Long Terminal Repeat (LTR) for an ERVL endogenous retrovirus (TguERVL2a2_LTR-La_fAlb) was found by searching in Dfam using *Ficedula albicollis* dataset as a songbird representative.

**Supplementary Figure 2: Intron size variation in reptile *CEACAM1* genes.** The position of *CEACAM1* exons on whole-genome shotgun contigs (WGS) were identified using BLAST with exon or cDNA nucleotide sequences as probes. Note the relatively small intron sizes in *Archelosauria* comprising alligator, birds and turtles and much larger intron sizes in *Squamata* including lizards, geckos and snakes. 5’UTR, 3’UTR, 5’-, 3’-untranslated regions; A, B, exon encoding IgC domains of subtype A, B; C1, CEACAM1; Cyt, cytoplasmic domains encoding exons; N, IgV type N domain; TM, transmembrane domain.

**Supplementary Figure 3 - CEACAM domain organization in different reptile clades.** Domain structure of bird (A), crocodile (B), turtle (C), lizard (D), gecko (E) and snake (F) CEACAMs. The domain organization of CEACAMs from selected reptile species was predicted by gene analysis. Family and species names are indicated at the left side. Domain organizations were confirmed by expression data from TSA data bases when available. IgV-like domains are shown as red, and IgC-like domains as blue ovals. ITIM and ITSM motifs are indicated by red and yellow, ITAM and ITAM-like endocytic motifs by green and blue boxes, respectively. CEACAMs with closely related IgV domains possibly representing paired receptors are connected by brackets. In the common wall lizard, a recent quintuplication of an A, B exon pair as identified by CLUSTALW analysis is indicated. C, CEACAM; TSA, Transcriptome Shotgun Assembly.

**Supplementary Figure 4 – Disintegrin-like motifs in snake CEACAM N and human PSG N domains.** (A) To identify putative disintegrin-like motives amino acid sequences of IgV-like N domain exons of predicted soluble snake CEACAM1L1, CEACAM1L2 and CEACAML3 and for comparison of membrane anchored CEACAM1 were aligned using the KALIGN multiple protein sequence alignment program. Amino acids identical in all sequences are shown in red (*), conserved amino acids in green (:), less conserved amino acids in blue (.). Consensus disintegrin motifs (RGD) are marked with dark gray underlay, less similar motifs (KGD, KGE, RGE) are labeled with a lighter gray color. For comparison, mature human PSG N domain alignments are shown. Location of the RGD motif in 3D models of the N domains of human PSG7 (B) and timber rattlesnake (*Crotalus horridus*, Cho) CEACAM1L1 (C). 3D model prediction was performed on the AlphaFold 3 server and visualized with ChimeraX. The predicted surface area of the RGD motif is shown in red. The GFC β-sheet consisting of β-strands G, F, C, C’ and C’’ faces the viewer at the leftmost orientation.

**Supplementary Figure 5 - Identification of *CEACAM1* genes in bird genera.** A total of 374 bird genera genomic databases were analyzed for the presence of *CEACAM1* exons encoding N, IgC-like domains A and B type, transmembrane domains (TM) and cytoplasmic domains (Cyt1–3ITIM/ITSM) using the CLUSTALW program and N, A, B, TM and Cyt1–3 exon sequences. A total of 199 genera are included in the phylogenetic tree with 185 genera found in the analyzed National Center of Biotechnology Information (NCBI) nucleotide sequence databases. Genera without circle could not be identified in the databases. The presence of complete CEACAM1 genes is indicated by light blue circles. CEACAM1 genes with gaps or missing exons are marked with purple, CEACAM1 pseudogenes with brown circles. The presence of CEACAM1 N exons with open reading frames is indicated by filled-in red circles, absence of N exons in database by open red circles. In a total of 54 genera one or more CEACAM1 exons could be identified. In 51 out of 185 analyzed bird genomes (27.6%) intact CEACAM1 N exons were found. Note, that some bird clades (like song birds (Passeriformes) and Columbaves) lack CEACAM1 genes. The phylogenetic tree was modified from Prum et al., 2015^51^.

**Supplementary Figure 6 – Alignment of CEACAM1 N exon nucleotide sequences from various chicken breeds reveals multiple insertions and deletions leading to pseudogene formation.** Chicken breed CEACAM1 N exon nucleotide sequences were identified using the N exon nucleotide sequence from the black-legged seriema (Chunga burmeisteri) and the NCBI blastn tool. Alignment was performed with the CLUSTALW multiple alignment program. Nucleotides Identical in all breeds are shown in in red, deletions are indicated by dashes. P, pseudogene.

**Supplementary Figure 7 - Expansion of extracellular domains of CEACAM1 in lizards**. Domain organization of selected lizard species were deduced from genomic data. Phylogenetic analyses revealed a possible scenario of an A, B exon pair duplication event during evolution: based on similarities light blue bars indicate older duplication events, while purple bars indicate more recent amplification events. Putative decoy (paired) receptor pairs with their N exon nucleotide sequence identity values (%) are marked by brackets. Species family, common and Latin names are indicated at the bottom.

**Supplementary Figure 8 - CEACAM1 splice variants in Western rattle snake.** Analysis of the Transcriptome Shotgun Assembly (TSA) database yielded multiple differentially spliced *CEACAM1* transcripts from the venom gland of the Western rattle snake. The *CEACAM1* transcripts differed in the number of spliced-in IgC-type exons and the presence of absence of a 53 bp cytoplasmic domain exon (Cyt1) which led to the formation of long form (L) and short (S) cytoplasmic domain splice variants, respectively.

**Supplementary Figure 9 - Reptile CEACAMs with ITAM-signaling motifs contain additional putative ITIM and endocytic motifs in their cytoplasmic domains.** The amino acid sequences of cytoplasmic domains of selected reptile species and for comparison human CEACAM3 and CEACAM4 were aligned using the KALIGN multiple protein sequence alignment program. Amino acids identical in all sequences are shown in red, conserved amino acids in green, less conserved amino acids in blue. The position and phases (0, 1, 2) of the introns are indicated. Please note, that the reptile CEACAM ITAM-containing cytoplasmic domains are encoded by 3, the human CEACAMs ITAM-containing cytoplasmic domains by 4 exons. Consensus sequences for ITAM, ITIM and endocytic motifs are indicated at the bottom^52–54^. For the three-letter abbreviation for species names see Supplementary Table 1.

**Supplementary Figure 10 – Selection for *CEACAM* N exon diversification and conservation in lizards.** (A) Diversification of N exon regions of putative pathogen CEACAM1 receptor genes between common wall and sand lizards was analyzed using the PipMaker program (gene names and species acronyms are shown in the lower right corner of the plot; please note that Lag_CEACAM1L5 represents the CEACAM1 ortholog in sand lizard). For contiguous stretches of nucleotides conserved between gene pairs, the degree of identity was calculated and displayed as horizontal black lines. The location of exons of the gene listed on top of the graphs is indicated by numbered boxes (red, IgV domain-encoding N exons; blue, IgC domain-encoding exons, black, transmembrane and cytoplasmic domain exons). Repeat sequences/mobile elements are shown by differently gray colored and shaped forms. For comparison, the level of conservation of the N exon regions is marked by red dashed lines, that of the most conserved IgC exon by blue lines. Red- and blue-outlined boxes indicate N exon- and C exon-encompassing regions of common identity. (B) Conservation of N exon regions of between CEACAM1 and the putative pathogen CEACAM1L1 decoy receptor gene in the common wall lizard was analyzed as described in (A). Lag, *Lacerta agilis* (sand lizard); Pmu, *Podarcis muralis* (common wall lizard).

**Supplementary Figure 11 – CEACAM N domain amino acid sequence identity and putative gene conversion event between reptile CEACAM paired receptors.** (A) The amino acid sequences of CEACAM1 and the closest ITAM-containing CEACAM from selected reptile species were compared using the KALIGN multiple protein sequence alignment program. Amino acids identical in all sequences are shown in red (*), conserved amino acids in green (:), less conserved amino acids in blue (.). The degree of identity is indicated in % below the alignments. Please note the high similarity between reptile CEACAM1 N domain amino acid sequences and putative decoy receptors exceeding that of an established decoy receptor (CEACAM3) in humans. (B) Nucleotide sequence alignment of central bearded dragon *CEACAM1* and *CEACAM1L2* N exons and flanking sequences was performed using the KALIGN multiple nucleotide sequence alignment program. Identical nucleotides are shown in red (*). Splice acceptor and splice donor sequences are highlighted in yellow and blue, respectively. Intron sequences are shown in lower case letters. For the three-letter abbreviation for species names see Supplementary Table 1.

## Data Availability

The nucleotide sequences of the reptile CEACAM N exons have been uploaded to the Zenodo open depository and can be retrieved using the Digital Object Identifier (DOI) https://zenodo.org/records/18457828?preview=1&token=eyJhbGciOiJIUzUxMiJ9.eyJpZCI6IjU0OThmZTMxLTYxYTktNGIxZi04ZWEyLWM0YmUxZjIwMDJlNCIsImRhdGEiOnt9LCJyYW5kb20iOiJlMzQzYjQ4NjdiODJlOGQwMjJhMzZjNTU2YzA4ODM5NSJ9.yix2tzktwO6dzwJ8ABnORz1V7cvWypEzZeFMKAaF8RlWTQgjr2EMvOMUrh5gMOr5onOApkQmM0mWXPPR0P3lDg.

## Abbreviations

CEA: carcinoembryonic antigen
CEACAM: CEA-related cell adhesion molecule
dN: number of nonsynonymous substitutions per nonsynonymous site
dS: number of synonymous substitutions per synonymous site
GPI: glycosylphosphatidylinositol
Ig: immunoglobulin-like
IgC: immunoglobulin constant-like domain
IgV: immunoglobulin variable-like domain
ITAM: immunoreceptor tyrosine-based activation motif
ITIM: immunoreceptor tyrosine-based inhibitory motif
ITSM: immunoreceptor tyrosine-based switch motif
N: N-terminal
PSG: pregnancy-specific glycoprotein
TSA: Transcriptome Shotgun Assembly
WGS: whole genome shot-gun
